# Hybracter: Enabling Scalable, Automated, Complete and Accurate Bacterial Genome Assemblies

**DOI:** 10.1101/2023.12.12.571215

**Published:** 2023-12-13

**Authors:** George Bouras, Ghais Houtak, Ryan R. Wick, Vijini Mallawaarachchi, Michael J. Roach, Bhavya Papudeshi, Lousie M. Judd, Anna E. Sheppard, Robert A. Edwards, Sarah Vreugde

**Affiliations:** 1 Adelaide Medical School, Faculty of Health and Medical Sciences, The University of Adelaide, Adelaide, Australia.; 2 The Department of Surgery - Otolaryngology Head and Neck Surgery, University of Adelaide and the Basil Hetzel Institute for Translational Health Research, Central Adelaide Local Health Network, South Australia, Australia.; 3 Department of Microbiology and Immunology, University of Melbourne at the Peter Doherty Institute for Infection and Immunity, Melbourne, Australia.; 4 Flinders Accelerator for Microbiome Exploration, College of Science and Engineering, Flinders University, Adelaide, Australia.; 5 Adelaide Centre for Epigenetics and South Australian Immunogenomics Cancer Institute, The University of Adelaide, Adelaide, Australia.; 6 School of Biological Sciences, The University of Adelaide, Adelaide, Australia.

## Abstract

Improvements in the accuracy and availability of long-read sequencing mean that complete bacterial genomes are now routinely reconstructed using hybrid (i.e. short- and long-reads) assembly approaches. Complete genomes allow a deeper understanding of bacterial evolution and genomic variation beyond small nucleotide variants (SNVs). They are also crucial for identifying plasmids, which often carry medically significant antimicrobial resistance (AMR) genes. However, small plasmids are often missed or misassembled by long-read assembly algorithms. Here, we present Hybracter, method for fast, automatic and scalable recovery of near-perfect complete bacterial genomes using a long-read first assembly approach. We compared Hybracter to existing automated hybrid assembly tools using a diverse panel of samples with manually curated ground truth reference genomes. We demonstrate that Hybracter is more accurate and faster than the existing gold standard automated hybrid assembler Unicycler. We also show that Hybracter with long-reads only is comparable to hybrid methods in recovering small plasmids.

## Introduction

Reconstructing complete bacterial genomes using *de novo* assembly methods had been considered too costly and time-consuming to be widely recommended in most cases, even as recently as 2015 ^1^. This was due to the reliance on short-read sequencing technologies, which does not allow for reconstructing regions with repeats and extremely high GC content ^2^. However, since then, advances in long-read sequencing technologies have allowed for the automatic construction of complete genomes using hybrid assembly approaches. Originally, this involved starting with a short-read assembly followed by scaffolding the repetitive and difficult to resolve regions with long-reads ^3,4^. This approach was implemented in the command-line tool Unicycler, which remains the most popular tool for generating complete bacterial genome assemblies ^5^. As long-read sequencing has improved in accuracy and availability, with the latest Oxford Nanopore Technologies reads recently reaching Q20 (99%+) median accuracy, a long-read first assembly approach supplemented by short-read polishing has recently been favoured for recovering accurate complete genomes. Long-read-first approaches provide greater accuracy and contiguity than short-read-first approaches in difficult regions ^6–11^. The current gold standard tool Trycycler even allows for the potential recovery of perfect genome assemblies ^7^. However, Trycycler requires significant microbial bioinformatics expertise and involves manual decision making, creating a significant barrier to useability, scalability and automation ^12^.

Several tools exist that generate automated long-read first genome assemblies, such as MicroPIPE ^13^, ASA3P ^14^, Bactopia ^15^ and Dragonflye ^16^. However, these tools do not consider factors such as genome reorientation ^17^ and recent polishing best-practices ^18^, and often contain the assembly workflow as a sub-module within a more expansive end-to-end pipeline. Additionally, none of the existing tools consider the targeted recovery of plasmids. As long-read assemblers struggle particularly with small plasmids, this leads to incorrectly recovered or missing plasmids in bacterial assemblies ^19^.

We introduce Hybracter, a new command-line tool for automated near-perfect long-read-first complete bacterial genome assembly. It implements a comprehensive and flexible workflow allowing for long-read assembly polished with long and short-reads (‘hybracter hybrid’ and ‘hybracter hybrid-single’) or long-read only assembly polished with long-reads (‘hybracter long’ and ‘hybracter long-single’). For ease of use and familiarity, Hybracter has been designed with a command-line interface containing parameters similar to Unicycler. Additionally, thanks to its Snakemake ^20^ and Snaketool ^21^ implementation, Hybracter seamlessly scales from a single isolate to hundreds or thousands of genomes with high computational efficiency and supports deployment on HPC clusters and cloud-based environments.

## Results

### Assembly Workflow

**Figure 1:**
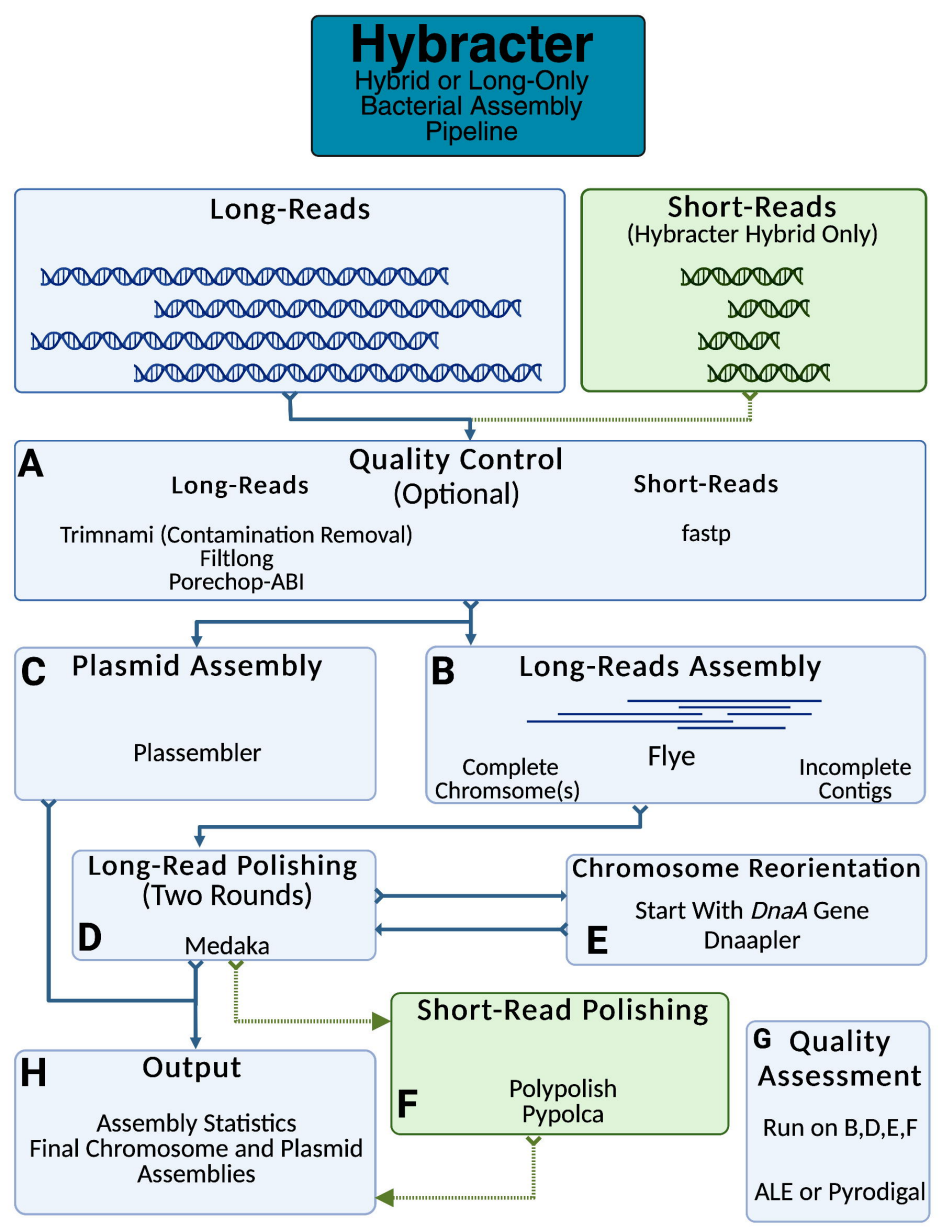
Outline of the Hybracter workflow.

Hybracter implements a long-read-first automated assembly workflow based on current best practices ^12^. The main subcommands available in Hybracter can be found in Table 1 and the workflow is outlined in Figure 1. Hybracter begins with long-reads for all subcommands, and with short-reads for polishing for ‘Hybracter hybrid’ and ‘Hybracter hybrid-single’ subcommands.

First, long-read input FASTQs are optionally filtered for quality control with Filtlong ^22^ and Porechop_ABI ^23^, with optional contaminant removal against a host genome using modules from Trimnami (e.g. if the bacteria has been isolated from a host) ^24^. Quality control of short-read input FASTQs is performed with fastp ^25^ (Fig 1A). Long-reads are then assembled with Flye ^26^. If at least 1 contig is recovered above the cut-off ‘-c’ chromosome length specified by the user for the sample, that sample will be denoted as ‘complete’. All such contigs will then be marked as chromosomes and kept for downstream reorientation and polishing. If zero contigs are above the cut-off chromosome length, the assembly will be denoted as ‘incomplete’, and all contigs will be kept for downstream polishing (Fig 1B).

For all complete samples, targeted plasmid assembly is then conducted using Plassembler ^27^ (Fig 1C). All assemblies are then polished with Medaka, which can be turned off using ‘--no_medaka’ ^28^ (Fig 1D). For complete assemblies, the chromosome will be reoriented to begin with the dnaA chromosomal replication initiator gene using Dnaapler ^29^. These chromosomes are then polished for a second time with Medaka to ensure the sequence around the original chromosome breakpoint is polished. If the user has provided short-reads using Hybracter hybrid, the assemblies are then polished with Polypolish ^18^ followed by pypolca ^30 31^ (Fig 1F). If short-reads are available (Hybracter hybrid), the quality of each assembly round is scored using ALE ^32^. If only long-reads are available (Hybracter long), the mean coding sequence (CDS) length is calculated for each assembly using Pyrodigal, with larger mean CDS lengths indicating a better quality assembly ^33 34^. The assembly with the highest mean CDS length is chosen.

Ultimately, the highest-scoring assembly is chosen as the final assembly (Fig 1G). A final output assembly FASTA file is created, along with per contig and overall summary statistic TSV files for each sample, and separate chromosome and plasmid FASTA files for samples denoted as complete (Fig 1H). Once the final assembly has been chosen for all samples, an overall ‘hybracter_summary.tsv’ file is generated. All main output files are explained in more detail in Table 2. While all these main outputs can be found in the ‘FINAL_OUTPUT’ subdirectory, all other intermediate output files are available for users who would like extra information about their assemblies, including all assembly assessments, comparisons of all changes introduced by polishing, and Flye and Plassembler output summaries. A full list of these supplementary outputs can be found in Hybracter’s Documentation (https://hybracter.readthedocs.io/en/latest/output/ ).

### Tool Selection

Tools were selected for inclusion in Hybracter either based on benchmarking from the literature, or they were specifically developed for inclusion in Hybracter. Flye ^26^ was chosen as the long-read assembler because it is more accurate than other long-read assemblers with comparable runtimes, such as Raven ^35^, Redbean ^36^ and Miniasm ^37^, while being dramatically faster than the comparably accurate Canu ^6,38^. Medaka ^28^ was chosen as the long-read polisher because of its ability to improve assembly continuity in addition to accuracy ^12,39^. The benchmarking results of this study also emphasise that it is particularly good at fixing insertion and deletion (InDel) errors, which cause problematic frameshifts and frequently lead to fractured or truncated gene predictions. Polypolish ^18^ and POLCA ^31^ were selected as short-read polishers, as these have been shown to achieve the highest performance with the lowest chance of introducing errors when used in combination ^18^.

We developed three standalone programs included in Hybracter. These are Dnaapler, Plassembler and Pypolca. Dnaapler was developed to ensure the chromosome(s) identified by Hybracter is reoriented to consistently begin with the dnaA chromosomal replication initiator gene. Full implementation details can be found in the manuscript, with expanded functionality beyond this use case ^29^. Plassembler was developed to improve the runtime and accuracy when assembling plasmids in bacterial isolates. Full implementation details can be found in the manuscript for hybrid mode ^27^. Hybracter long utilises Plassembler containing a post-publication improvement for long-reads only (‘Plassembler long’) released in v1.3. Plassembler long assembles plasmids from only long-reads by treating long-reads as both short-reads and long-reads. Plassembler long does this by utilising Unicycler in its pipeline to create a de Bruijn graph-based assembly, treating the long-reads as unpaired single-end reads, which are then scaffolded with the same long-read set.

The third tool is Pypolca. Pypolca is a Python re-implementation of the POLCA short-read genome polisher, created specifically for inclusion in Hybracter and with an almost identical output format and performance (see Methods). Compared to POLCA, Pypolca features improved useability with a simplified command line interface and allows the user to specify an output directory. Furthermore, Pypolca is available on both MacOS and Linux (POLCA is only available on Linux) and does not require the installation of the entire MaSuRCA genome assembler toolkit ^40^. Pypolca is open-source and freely available on Bioconda, PyPI, and GitHub (https://github.com/gbouras13/pypolca).

### Benchmarking

To test the performance of Hybracter, we used 20 samples with available short- and long-read sets. These samples represent genomes from a variety of Gram-negative and Gram-positive bacteria, with most containing plasmids. We chose these samples as they have both real hybrid read sets and manually curated genome assemblies produced using either Trycycler ^7^ or Bact-builder ^41^—a consensus-building pipeline based on Trycycler. We tested Hybracter with both short- and long-reads (‘Hybracter hybrid’) and long-reads only (‘Hybracter long’) against Unicycler and the Dragonflye ^16^ pipeline both with long-reads only (‘Dragonflye long’) and with short-read polishing ( ‘Dragonflye hybrid’). More benchmarking details can be found in the Methods section.

### Chromosome Accuracy Performance

All tools recovered complete circular contigs for each chromosome. SNVs, small InDels (under 60 bps), and large InDels (over 60 bps) were compared as a measure of assembly accuracy. To account for differences in genomic size between isolates, SNVs and small InDel counts were normalised by genome length.

**Figure 2:**
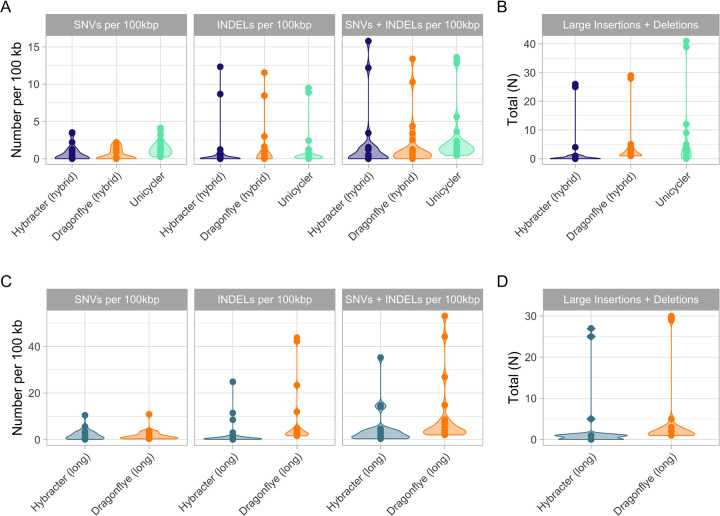
Comparison of the counts of small nucleotide variants (SNVs) and small (<60bp) insertions and deletions (InDels) per 100kbp (A) and the total number of large (>60bp) InDels (B) for the Hybrid tools benchmarked (Hybracter hybrid in blue, Dragonflye hybrid, in orange and Unicycler in green). The counts of SNVs and small InDels per 100kbp (C) and the total number of large InDels (D) for the long tools benchmarked (Hybracter long in blue, Dragonflye long in orange) are also shown. All data presented is from the benchmarking output run with 8 threads.

The summary results are presented in Table 3 and visualised in Figure 2. The detailed results for each tool and sample are presented in Supplementary Table 5. Of the hybrid tools, Dragonflye hybrid produced the fewest SNVs per 100kbp (median 0.03) followed by Hybracter hybrid (median 0.16) and Unicycler (median 1.25). Hybracter hybrid produced the fewest InDels per 100kbp (median 0.05), followed by Unicycler (median 0.28) and Dragonflye hybrid (median 0.49). Hybracter hybrid also produced the fewest InDels plus SNVs per 100kbp (median 0.24), followed by Dragonflye hybrid (median 0.74) and Unicycler (median 1.49). The median InDels plus SNVs per 100kbp rate for Hybracter is very low, with 0.24 small InDels plus SNVs per 100kbp corresponding to approximately 12 small InDels plus SNVs total for a standard 5MB *E. coli* genome.

Additionally, Hybracter hybrid showed superior performance in terms of large InDels, with a median of 0 and a total of 59 large InDels across the 20 samples, compared to 1.5 and 91 for Dragonflye hybrid, and 2.5 and 134 for Unicycler.

Overall, Hybracter hybrid produced the most accurate chromosome assemblies. For eight isolates described in *Lerminiaux* et al. ^9^, Hybracter also assembled a perfect chromosome (Isolates A, B, C, D, E, G, I, L), and another two near-perfect chromosomes (defined as <10 total SNVs plus InDels) (Isolate K and H37R2). Dragonflye hybrid did not assemble any perfect chromosomes and recovered six near-perfect chromosomes (Isolate B, D, E, G, H, I).

Similar results were found in the long-read only tool comparison. Dragonflye long produced slightly fewer SNVs per 100kbp (median = 0.99) than Hybracter long (median 1.07). However, Hybracter long consistently had fewer small InDels (median 0.49), large InDels (total 66) and small InDels plus SNVs per 100kbp (median 2.08) than Dragonflye long (median 3.01, total 92 and median 3.81 respectively). No perfect or near-perfect chromosomes were assembled by either long-only tool, though Hybracter long did assemble several chromosomes with fewer than 50 total small InDels plus SNVs (*Lerminiaux* isolates A, C, D, G, H, L, J, and ATCC BAA-679).

Additionally, long-read only assembly methods had consistently worse performance than hybrid tools as measured by SNVs and small InDels, suggesting the continuing utility of short-read polishing for the isolates surveyed.

### Plasmid Recovery Performance and Accuracy

Hybracter in both hybrid and long modes was superior at recovering plasmids compared to the other tools in the same class (Table 4). Hybracter hybrid was able to completely recover 57/59 possible plasmids (the other two were partially recovered), compared to 54/59 for Unicycler and only 34/59 for Dragonflye hybrid. Hybracter hybrid did not miss a single plasmid, while Unicycler missed 3/59 (all in Isolate E from *Lerminiaux* et al. ^9^) and Dragonflye hybrid completely missed 9/59. In terms of accuracy, Hybracter hybrid and Unicycler were similar in terms of SNVs plus small InDels, with medians of 4.15 and 3.83 per 100kbp respectively (Supplementary Table 9), while Hybracter hybrid produced fewer large InDels than Unicycler (39 vs 51 in total).

Interestingly, Hybracter long showed strong performance at recovering plasmids despite using only long-reads, completely recovering 54/59 plasmids, completely missing only 2/59. This performance was far superior to Dragonflye long (35/59 completely recovered, 8/59 missed). In terms of accuracy, both long tools were similar and unsurprisingly less accurate than the hybrid tools in terms of SNVs plus small InDels (medians of 10.64 per 100kbp for Hybracter long and 9.22 per 100kbp for Dragonflye long). However, Hybracter long was the best-performing tool overall as measured by large InDels (total 32), outperforming all hybrid tools and dragonflye long (total 123). Additionally, all five tools detected an additional 5411bp plasmid in *Lerminiaux* Isolate G not found in the reference sequence and Hybracter in both hybrid and long modes detected a further 2519bp small plasmid from this genome.

Hybracter hybrid recovers more plasmids than either Unicycler or Dragonflye because it uses a dedicated plasmid assembler, Plassembler ^27^. In addition, Hybracter long, using only long-reads had an identical complete plasmid recovery rate to Unicycler, which uses both long- and short-reads (54/59 for both). These results suggest that Hybracter long, by applying algorithms designed for short-reads on long-reads, largely solves the existing difficulties of recovering small plasmids from long-reads, at least on the benchmarking dataset of predominantly R10 Nanopore reads ^19,42^.

Another interesting result from Hybracter hybrid is that in 6/20 isolates, it assembled additional non-plasmid contigs, which occurred in only 1/20 isolates for Unicycler. These contigs are not necessarily an assembly artifact and can provide additional information regarding the quality control and similarity of short and long-read sets. In Plassembler implemented within Hybracter hybrid, the existence of such contigs is often indicative of mismatches between long- and short-read sets ^27^, suggesting that there is likely some heterogeneity between long- and short-reads in those six samples.

### Runtime Performance Comparison

**Figure 3:**
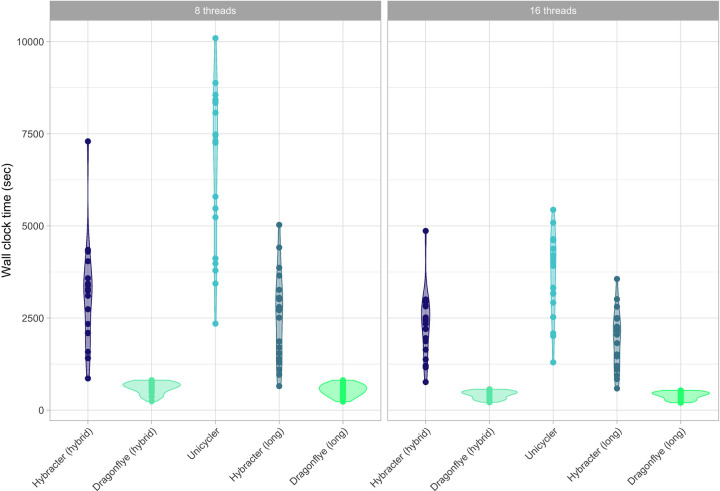
Comparison of wall-clock runtime (in seconds) of Hybracter hybrid, Dragonflye hybrid, Unicycler, Hybracter long and Dragonflye long when run with 8 and 16 threads.

As shown in Table 5 and Figure 3, median wall-clock times with 8 threads for Dragonflye hybrid (10m55s) were smaller than Hybracter hybrid (54m23s), which were in turn smaller than Unicycler (02h03m02s). For the long-only tools, Dragonflye long (9m24s) was faster than Hybracter long (45m29s). Hybracter long was consistently slightly faster than Hybracter hybrid (Table 5).

The difference in runtime performance between Hybracter and Dragonflye is predominantly the result of the included targeted plasmid assembly and the reorientation and assessment steps in Hybracter that are not included in Dragonflye. Additionally, the results suggest limited benefits to running Hybacter with more than eight threads. As explained in the following section, if a user has multiple isolates to assemble, a superior approach is to modify the configuration file specifying more efficient resource requirements for each job in Hybracter.

### Parallelisation Allows for Improved Efficiency

Hybracter allows users to specify and customise a configuration file to maximise resource usage and runtime efficiency. Users can modify the desired threads, memory and time requirements for each type of job that is run within Hybracter to suit their computational resources. So that resources are not idle for most users on single sample assemblies, large jobs such as the Flye and Plassembler assembly steps default to 16 threads and 32 GB of memory.

To emphasise the efficiency benefits of parallelisation, the 12 *Lerminiaux* et al. isolates were also assembled using ‘hybracter hybrid’ with a customised configuration file designed to improve efficiency on the machine used for benchmarking. Specifically, the configuration was changed to specify 8 threads and 16 GB of memory allocated to big jobs (assembly, polishing and assessment) and 4 threads and 8 GB of memory allocated to medium jobs (reorientation). More details on changing Hybracter’s configuration file to suit specific systems can be found in the documentation (https://hybracter.readthedocs.io/en/latest/configuration/). We limited the overall ‘hybracter hybrid’ run with 32 GB of memory and 16 threads to provide a fair comparison. The overall ‘hybracter hybrid’ run was then compared to the sum of the 12 ‘hybracter hybrid-single’ runs. Overall, the 12 isolates took 06h16m53s in the combined run, as opposed to 09h34m08s from the sum of the 12 ‘hybracter hybrid-single’ and 13h32m51s from the sum of the 12 Unicycler runs. This inbuilt parallelisation of Hybracter provides significant efficiency benefits if multiple samples are assembled simultaneously. The performance benefit of Hybracter afforded by Snakemake integration in parallel computing systems may be variable over different architectures, but this provides an example case of potential efficiency and convenience benefits.

## Discussion

As long-read sequencing has improved in accuracy with reduced costs, it is now routine to use a combination of long- and short-reads to generate complete bacterial genomes ^3,5^ Recent advances in assembly algorithms and accuracy improvements mean that a long-read first hybrid assembly should be favoured with short-reads being used after assembly for polishing ^12^, as opposed to the short-read first assembly approach (where long-reads are only used for scaffolding a short-read assembly) utilised by the current automated gold standard Unicycler. The Unicycler approach is more prone to larger scale InDel errors as well as smaller scale errors such as those caused by homopolymers or methylation motifs ^6,11,43,44^. Additionally, it should be noted that it is already possible (while perhaps not routine) to generate perfect hybrid bacterial genome assemblies using manual consensus approaches requiring human intervention, such as Trycycler ^7,45^. While manual approaches such as Trycycler generally yield superior results to automated approaches, manually assembling many complete genomes manually is challenging as considerable time, resources and bioinformatics expertise are required.

The results of this study emphasise that the long-read first hybrid approach consistently yields superior assemblies than the short-read first hybrid approach and should therefore be preferred going forward. This study also shows that automated perfect hybrid genome assemblies are already possible with Hybracter. This study and others ^9,46^ also confirm that a long-read first hybrid approach remains preferable to long-read only assembly with Nanopore reads, as short-reads continue to provide accuracy improvements in polishing steps. However, it is foreseeable that short-reads will provide little or no accuracy improvements and will not be needed to polish long-read only assemblies; perfect long-read only assemblies are already possible, at least with manual intervention using Trycycler ^11^. Accordingly, automated perfect (or near-perfect) bacterial genome assemblies may soon become possible from long-reads only. Hybracter maintains the flexibility to use long-reads only if desired, allowing users to turn long-read polishing all-together. This may become increasingly useful as long-read sequencing continues to improve in accuracy beyond the read sets used in this study, because long-read polishing can introduce errors and make long-read only assemblies worse with highly accurate Nanopore and PacBio reads ^8,11^.

Hybracter was created to bridge the gap from the present to the future of automated perfect hybrid and long-read-only bacterial genome assemblies. The results of this study show that Hybracter in hybrid mode is both faster and more accurate than the current gold standard tool for hybrid assembly Unicycler and is more accurate than Dragonflye in both modes. It should be noted that if users want fast chromosome only assemblies where accuracy is not essential (for applications such as species identification or sequence typing), Dragonflye remains a good option due to its speed.

Hybracter especially excels in recovering complete plasmid genomes compared to other tools. By incorporating Plassembler, Hybracter recovers more complete plasmid genomes than Unicycler in hybrid mode. Further, Hybracter long is comparable to Unicycler and Hybracter hybrid when using long-reads only for plasmid recovery.

The high error rates of long-read sequencing technologies have prevented the application of assembly approaches designed for highly accurate short-reads, such as constructing de Bruijn graphs (DBGs) based on strings of a particular length *k* (*k*-mers)^47–49^. This resulted in bioinformaticians initially utilising less efficient algorithms designed with long-reads in mind, such as utilising overlap graphs in place of DBGs ^26,36,38,50,51^. While DBGs have been used for long-read assembly in some applications ^52–54^, adoption, especially in microbial genomics, has been limited.

Although long-read first assembly methods enable complete chromosome and large plasmid reconstruction, it is well established that long-read only assemblers struggle to assemble small (<20kbp) plasmids accurately, often leading to missing or multiplicated assemblies^19,27^. These errors may be exacerbated if ligation chemistry based sequencing kits are used^42^. Therefore, hybrid DBG based short-read first assemblies are traditionally recommended for plasmid recovery ^12^.

Implemented in our post-publication changes to Plassembler described in this study, Hybracter solves the problem of small plasmid recovery using long-reads. It achieves this by implementing a DBG-based assembly approach with Unicycler. The same read set is used twice, first as unpaired pseudo ‘short’ reads and then as long-reads; the long-read set scaffolds a DBG-based assembly based on the same read set. This study demonstrates that current long-read technologies, such as R10 Nanopore reads, are now accurate enough that some short-read algorithms are applicable. Our results also suggest that similar DBG-based algorithmic approaches could be used to enhance the recovery of small replicons in long-read datasets beyond the use case presented here of plasmids in bacterial isolate assemblies. This could potentially enhance the recovery of replicons such as bacteriophages ^55^ or other small contigs from metagenomes using only long-reads ^10,56^.

Finally, consistent and resource efficient assemblies that are as accurate as possible in recovering both plasmids and chromosomes are crucial, particularly for larger studies investigating plasmid epidemiology and evolution. AMR genes carried on plasmids can have complicated patterns of transmission involving horizontal transfer between different bacterial species and lineages, transfer between different plasmid backbones, and integration into and excision from the bacterial chromosome ^57–59^. Accurate plasmid assemblies are crucial in genomic epidemiology studies investigating transmission of antimicrobial resistant bacteria within outbreak settings, as well as in a broader One Health context, where hundreds or even thousands of assemblies may be analysed ^60–63^. Hybracter will facilitate the expansion of such studies, allowing for faster and more accurate automated complete genome assemblies than existing tools. Additionally, by utilising Snakemake^20^ with a Snaketool^21^ command line interface, Hybracter is easily and efficiently parallelised to optimise available resources over various large-scale computing architectures. Individual jobs (such as each assembly, reorientation, polishing or assessment step) within Hybracter are automatically sent to different resources on a high performance computing (HPC) cluster using the HPC’s job scheduling system like Slurm^64^. Hybracter can natively use any Snakemake-supported cloud-based deployments such as Kubernetes, Google Cloud Life Sciences, Tibanna, and Azure Batch.

## Conclusion

Hybracter is substantially faster than the current gold standard tool Unicycler, assembles chromosomes more accurately than existing methods, and is superior at recovering complete plasmid genomes. By applying DBG-based algorithms designed for short-reads on current generation long-reads, Hybracter long also solves the problem of long-read-only assemblers entirely missing or duplicating small circular elements such as plasmids. Hybracter is resource efficient and natively supports deployment on high-performance computer clusters and cloud environments for massively parallel analyses. We believe Hybracter will prove to be an extremely useful tool for the automated recovery of complete bacterial genomes from hybrid and long-read-only sequencing data suitable for massive datasets.

## Methods

### Benchmarking

To compare Hybracter’s functionality and performance, we benchmarked its performance against other software tools. We focused on the most popular state-of-the-art assembly tools for automated hybrid and long only bacterial genome assemblies. All code to replicate these analyses can be found at the repository (https://github.com/gbouras13/hybracter_benchmarking ). All programs and dependency versions used for benchmarking can be found in Supplementary Table 4. For the hybrid tools, we chose Unicycler and Dragonflye with both long-read and short-read polishing (denoted ‘Dragonflye hybrid’). Dragonflye was chosen as it is a popular long-read first assembly pipeline ^16^. Both tools were run using default parameters. By default, Dragonflye conducts a long-read assembly with Flye that is polished by Racon ^65^ followed by Polypolish. For the long-read only tool, we chose Dragonflye with long-read Racon based polishing only (denoted ‘Dragonflye long’).

We used 20 samples for benchmarking, representing genomes from a variety of Gram-negative and Gram-positive bacteria. We chose these samples as they have real hybrid read sets in combination with manually curated genome assemblies produced using either Trycycler or Bact-builder ^41^—a consensus-building pipeline based on Trycycler. These samples came from 4 different studies below. We used the published genomes from these studies (or the available genomes available from the ATCC) as representatives of the ‘ground truth’ for these samples. Where read coverage exceeded 100x samples were subsampled to approximately 100x coverage of the approximate genome size with Rasusa v0.7.0 ^66^, as this better reflects more realistic read depth of real life isolate sequencing. Nanoq v0.10.0 ^67^ was used to generate quality control statistics for the subsampled long-read sets. Four isolates did not have 100x long-read coverage — the entire long-read set was used instead. A full summary table of the read lengths, quality, Nanopore kit and base-calling models used in these studies can be found in Supplementary Table 2.

These samples contained varying levels of long-read quality (reflecting improvements in Oxford Nanopore Technologies long-read technology), with the median Q score of long-read sets ranging from 12.3 to 18.3. The four studies are:

1. Five ATCC strain isolates (ATCC-10708 *Salmonella enterica*, ATCC-17802 *Vibrio paragaemolyticus*, ATCC-25922 *Escherichia coli*, ATCC-33560 *Campylobacter jejuni* and ATCC-BAA-679 *Listeria monocytogenes* ) made available as a part of this study ^8^
2. Twelve diverse carbapenemase-producing Gram-negative bacteria from *Lerminiaux* et al. ^9^
3. *Staphylococcus aureus* JKD6159 sequenced with both R9 and R10 chemistry long-read sets from *Wick* et al. ^45^
4. *Mycobacterium tuberculosis* HR37v from *Chitale* et al. ^41^

The full details for each individual isolate used can be found in Supplementary Tables 1 and 2.

### Chromosome Accuracy

The assembly accuracy of the chromosomes recovered by each benchmarked tool was compared using Dnadiff v1.3 packaged with MUMmer v3.23 ^68^. Comparisons were performed on the largest assembled contig (denoted as the chromosome) by each method, other than for ATCC-17802 *Vibrio parahaemolyticus*, where the two largest contigs were chosen as it has two chromosomes.

### Plasmid Recovery Performance and Accuracy

Plasmid recovery performance for each tool was compared using the following methodology. Summary statistics are presented considered in Table 4. See Supplementary Table 7 for a full sample-by-sample analysis. All samples were analysed using the 4-step approach outlined below using summary length and GC% statistics for all contigs and the output of Dnadiff v1.3 comparisons generated for each sample and tool combination against the reference genome plasmids:

1. The number of circularised plasmid contigs recovered for each isolate was compared to the reference genome. If the tool recovered a circularised contig homologous to that in the reference, it was denoted as completely recovered. Specifically, a contig was denoted as completely recovered if it had a genome length within 250bp of the reference plasmid, a GC% within 0.1% of the reference plasmid and whether the Total Query Bases covered was within 250bp of the Total Reference Bases from Dnadiff. For Dragonflye assemblies, some plasmids were duplicated or multiplicated due to known issues with the long-read first assembly approach for small plasmids ^6,19,42^. Any circularised contigs that were multiplicated compared to the reference plasmid were therefore denoted as misassembled.
2. For additional circularised contigs not found in the reference recovered, these were tested for homology with NCBI nt database using the web version of blastn ^69^. If there was a hit to a plasmid, the Plassembler output within Hybracter was checked for whether the contig had a Mash hit (i.e. a Mash distance of 0.2 or lower) to plasmids in the PLSDB ^70^. If there was a hit, the contig was denoted as an additional recovered plasmid. There were 2 in total (see Supplementary Table 7 and supplementary data).
3. Plasmids with contigs that were either not circularised but homologous to a reference plasmid, or circularised but incomplete (failing the genome length and Dnadiff criteria in 1.) were denoted as partially recovered or misassembled.
4. Reference plasmids without any homologous contigs in the assembly were denoted as missed.

Additional non-circular contigs that had no homology with reference plasmids and were not identified as plasmids in step 2 were analysed on a contig-by-contig basis and denoted as additional non-plasmid contigs (see Supplementary Table 7 for contig-by-contig analysis details).

### Runtime Performance Comparison

To compare the performance of Hybracter, we compared wall-clock runtime consumption on a machine with an Intel^®^ Core^™^ i7–10700K CPU @ 3.80 GHz on a machine running Ubuntu 20.04.6 LTS with a total of 16 available threads (8 cores). We ran all tools with 8 and 16 threads and with 32 GB of memory to provide runtime metrics comparable to commonly available consumer hardware. Hybracter hybrid and long were run with ‘hybracter hybrid-single’ and ‘hybracter long-single’ for each isolate to generate a comparable per sample runtime for comparison with the other tools. The summary results are available in Table 5 and the detailed results for each specific tool and thread combination are found in Supplementary Table 8.

### Sequencing

DNA extraction was performed with the DNeasy Blood and Tissue kit (Qiagen). Illumina library preparation was performed using Illumina DNA prep (Illumina Inc.) according to the manufacturer’s instructions. Short-read whole genome sequencing was performed an Illumin MiSeq with a 250bp PE kit. Oxford Nanopore Technologies library preparation ligation sequencing library was prepared using the ONT SQK-NBD114–96 kit and the resultant library was sequenced using an R10.4.1 MinION flow cell (FLO-MIN114) on a MinION Mk1b device. Data was base-called with Super-Accuracy Basecalling (SUP) using the basecaller model dna_r10.4.1_e8.2_sup@v3.5.1.

### Pypolca Benchmarking

Pypolca v0.2.0 was benchmarked against POLCA (in MaSuRCA v4.1.0) ^31^ using 18 isolates described above. These were all 12 *Lerminiaux* et al. isolates, the R10 JKD6159 isolate ^45^ and the 5 ATCC samples we sequenced as a part of this study. Benchmarking was conducted on an Intel^®^ Core^™^ i7–10700K CPU @ 3.80 GHz on a machine running Ubuntu 20.04.6 LTS. All short read FASTQs used for benchmarking are identical to those used to benchmark Hybracter. The assemblies used for polishing were intermediate chromosome assemblies from Flye v2.9.2 ^26^ generated within Hybracter. The outputs from Pypolca and POLCA were compared using Dnadiff v1.3 packaged with MUMmer v3.23 ^68^ Overall, Pypolca and POLCA yielded extremely similar results. 16/18 assemblies were identical. ATCC 33560 had 2 Single Nucleotide Polymorphisms (SNPs) between Pypolca and POLCA and *Lerminiaux* Isolate I also had 2 SNPs.

## Data Availability

The subsampled FASTQ files used for benchmarking are publicly available at Zenodo with DOI (https://doi.org/10.5281/zenodo.10158013 ). All ATCC FASTQ reads sequenced as a part of this study can be found under BioProject PRJNA1042815 with the genomes publicly available from the ATCC. All raw *Lermaniaux* et al. FASTQ read files and genomes (prior to subsampling) can be found in the SRA under BioProject PRJNA1020811. All *Staphylococcus aureus* JKD6159 FASTQ read files and genomes can be found under BioProject PRJNA50759. All *Mycobacterium tuberculosis* H37R2 FASTQ read files and genomes can be found under BioProject PRJNA836783. The complete list of BioSample accession numbers for each benchmarked sample can be found in Supplementary Table 1. The benchmarking assembly output files are publicly available on Zenodo with DOI (https://doi.org/10.5281/zenodo.10158013 ). All Pypolca benchmarking outputs and code are publicly available on Zenodo with DOI (https://zenodo.org/doi/10.5281/zenodo.10072192 ).

## Code Availability

Hybracter is developed using Python and Snakemake as a command-line software tool for Linux and MacOS systems. Hybracter is freely available under an MIT License on GitHub (https://github.com/gbouras13/hybracter) and the documentation is available at Read the Docs (https://hybracter.readthedocs.io/en/latest/). Hybracter is available to install via PyPI (https://pypi.org/project/hybracter/) and Bioconda (https://anaconda.org/bioconda/hybracter). All code used to benchmark Hybracter, including the reference genomes, is publicly available on GitHub (https://github.com/gbouras13/hybracter_benchmarking) with released DOI (https://zenodo.org/doi/10.5281/zenodo.10157987 ) available at Zenodo.

## Supplementary Material

Supplement 1

## Figures and Tables

**Table 1.** Summary of the 4 Primary Hybracter Commands

Command	Input	Number of Samples	Description	Workflow Elements Included by Default (From Figure 1)
***Hybracter hybrid***	5 column csv sample sheet specified with ‘--input’ containing: • sample name • long-read FASTQ path, • estimated chromosome length • R1 short-read FASTQ path • R2 short-read FASTQ path	1+	Long-read first assembly followed by long then short-read polishing for multiple isolates. Snakemake implementation ensures efficient use of available resources	A, B, C, D, E, F, G, H
***hybracter hybrid-single***	• sample name (-s) • long-read FASTQ path (-l) • estimated chromosome length (-c) • R1 short-read FASTQ path (−1) • R2 short-read FASTQ path (−2)	1	Long-read first assembly followed by long then short-read polishing for a single isolate. Similar command line interface to Unicycler	A, B, C, D, E, F, G, H
***hybracter long***	3 column csv sample sheet specified with ‘--input’ containing: • sample name • long-read FASTQ path, • estimated chromosome length	1+	Long-read first assembly followed by long-read polishing for multiple isolates. Snakemake implementation ensures efficient use of available resources	A (no fastp), B, C, D, E, G, H
***hybracter long-single***	• sample name (-s) • long-read FASTQ path (-l) • estimated chromosome length (-c)	1	Long-read first assembly followed by long-read polishing on a single isolate.	A (no fastp), B, C, D, E, G, H

**Table 2.** Description of the Primary Hybracter Output Files

Output File	Description
**{sample}_final.fasta**	Final assembly FASTA file for the sample. Contains all chromosome(s) and plasmids for complete isolates and all contigs for incomplete isolates.
**{final}_chromosome.fasta**	Final assembly FASTA file for the chromosomes(s) in a complete sample.
**{final}_plasmid.fasta**	Final assembly FASTA file for the plasmids in a complete sample.
**hybracter_summary.tsv**	A TSV file combining the {sample}_summary.tsv files for all samples.
**{sample}_summary.tsv**	A TSV file containing columns denoting for the sample: • Assembly completeness • Total assembly length • Number of contigs assembled • The polishing round deemed to be most accurate and selected as the final assembly • The length of the longest contig • The estimated coverage of the longest contig • The number of circular plasmids recovered by Plassembler
**{sample}_per_contig_stats.tsv**	A TSV file containing columns denoting for the sample: • Contig name • Contig Type (chromosome or plasmid) (complete samples only) • Contig length • Contig GC% • Contig circularity (complete samples only)

**Table 3.** Small (<60bp) InDels, SNVs per 100kbp of sequence and total large (>60bp) InDels of Chromosomes Assemblies for all Benchmarked Isolates.

Tool	Type	Small InDels per 100kbp	SNVs per 100kbp	Small InDels + SNVs per 100kbp	Large InDels
**Hybracter hybrid**	Hybrid	Median = 0.05 Minimum = 0 Maximum = 12.34	Median = 0.16 Minimum = 0 Maximum = 3.52	Median = 0.24 Minimum = 0 Maximum = 15.79	Total = 59 Median = 0 Minimum = 0 Maximum = 26
**Dragonflye hybrid**	Hybrid	Median = 0.49 Minimum = 0 Maximum = 11.56	Median = 0.03 Minimum = 0 Maximum = 2.21	Median = 0.74 Minimum = 0 Maximum = 13.41	Total = 91 Median = 1.5 Minimum = 1 Maximum = 29
**Unicycler**	Hybrid	Median = 0.28 Minimum = 0 Maximum = 9.5	Median = 1.25 Minimum = 0.25 Maximum = 4.13	Median = 1.49 Minimum = 0.43 Maximum = 13.62	Total = 134 Median = 2.5 Minimum = 0 Maximum = 41
**Hybracter long**	Long	Median = 0.49 Minimum = 0.06 Maximum = 24.82	Median = 1.07 Minimum = 0.07 Maximum = 10.46	Median = 2.08 Minimum = 0.37 Maximum = 35.29	Total = 66 Median = 1 Minimum = 0 Maximum = 27
**Dragonflye long**	Long	Median = 3.01 Minimum = 1.61 Maximum = 43.8	Median = 0.99 Minimum = 0.33 Maximum = 10.86	Median = 3.81 Minimum = 2.01 Maximum = 53.1	Total = 92 Median = 2 Minimum = 1 Maximum = 30

**Table 4.** The Total Number of Plasmids Recovered by Each Tool. There were 59 total reference plasmids in the 20 samples.

Tool	Complete Plasmids Recovered	Total Plasmids Partially Recovered or Misassembled	Total Plasmids Missed	Additional Plasmids Recovered not in Reference	Additional Non-Plasmid Contigs Recovered
**Hybracter hybrid**	57	2	0	2	6
**Unicycler**	54	2	3	1	1
**Dragonflye hybrid**	34	16	9	1	7
**Hybracter long**	54	3	2	2	3
**Dragonflye long**	35	16	8	1	5

**Table 5.** Wall-clock Runtime Summary Statistics for Each Tool.

Tool	Type	8 Threads (hh:mm:ss)	16 Threads (hh:mm:ss)
**Hybracter hybrid**	Hybrid	Median = 00:54:23 Minimum = 00:14:22 Maximum = 02:01:37	Median = 00:40:19 Minimum = 00:12:43 Maximum = 01:21:05
**Dragonflye hybrid**	Hybrid	Median = 00:10:55 Minimum = 00:04:02 Maximum = 00:13:34	Median = 00:07:21 Minimum = 00:03:33 Maximum = 00:09:28
**Unicycler**	Hybrid	Median = 02:03:02 Minimum = 00:39:09 Maximum = 02:48:16	Median = 01:06:8 Minimum = 00:21:38 Maximum = 01:30m38
**Hybracter long**	Long	Median = 00:45:29 Minimum = 00:10:52 Maximum = 01:23:49	Median = 00:34:56 Minimum = 00:09:49 Maximum = 00:59:21
**Dragonflye long**	Long	Median = 00:09:24 Minimum = 00:03:52 Maximum = 00:13:32	Median = 00:07:00 Minimum = 00:03:22 Maximum = 00:08:56
